# Medicines Related Problems (MRPs) Originating in Primary Care Settings in Older Adults - A Systematic Review

**DOI:** 10.1177/08971900211023638

**Published:** 2021-06-23

**Authors:** Rosetta Chinyere Ude-Okeleke, Zoe Aslanpour, Soraya Dhillon, Nkiruka Umaru

**Affiliations:** 1 School of Life and Medical Sciences, University of Hertfordshire, Hatfield, United Kingdom

**Keywords:** older persons, pharmaceutical care issues, primary health care, MRPs, geriatrics

## Abstract

**Background:**

As people age, they become increasingly vulnerable to the untoward effects of medicines due to changes in body systems. These may result in medicines related problems (MRPs) and consequent decline or deterioration in health.

**Aim:**

To identify MRPs, indicators of deterioration associated with these MRPs, and preventative interventions from the literature.

**Design and Setting:**

Systematic review of primary studies on MRPs originating in Primary Care in older people.

**Methods:**

Relevant studies published between 2001 and April 2018 were obtained from Medline (via PubMed), CINAHL, Embase, Psych Info, PASCAL, Scopus, Cochrane Library, Science Direct, and Zetoc. Falls, delirium, pressure ulcer, hospitalization, use of health services and death were agreed indicators of deterioration. The methodological quality of included studies was assessed using the Down and Black tool.

**Results:**

There were 1858 articles retrieved from the data bases. Out of these, 21 full text articles met inclusion criteria for the review. MRPs identified were medication error, potentially inappropriate medicines, adverse drug reaction and non-adherence. These were associated with indicators of deterioration. Interventions that involved doctors, pharmacists and patients in planning and implementation yielded benefits in halting MRPs.

**Conclusion:**

This Systematic review summarizes MRPs and associated indicators of deterioration. Appropriate interventions appeared to be effective against certain MRPs and their consequences. Further studies to explore deterioration presented in this systematic review is imperative.

## Introduction

Ensuring the safety of medication use in older people can often be challenging as older people often have several coexisting medical problems and take multiple drugs. Other reported, features that can be important to older people in terms of medicines handling are reduced mobility, reduced cognition, as well as increased frailty.^1^ Furthermore, body system changes that occur with aging can contribute to this difficulty in medicines handling.^2^ Central to health and care of some older people is the management of a variety of medical conditions commonly referred to as “geriatric syndrome” (GS) which can include fall, delirium, pressure ulcer and underfeeding.^3^ Sanchez et al (2011)^4^ reported a prevalence of 60.2% geriatric syndrome in older patients with acute cardiac disease. On the other hand, Nair, and Colleagues (2008)^5^ reported a GS prevalence of between 29% (cognitive impairment including delirium and dementia) and 54% (Fall) in older patients admitted to a tertiary hospital. This implies that GS is common in older people. This syndrome can develop when compensatory abilities of older people decline or become compromised by accumulated impairment in multiple areas of the body otherwise referred to as frailty.^6^ This syndrome can also be a consequence of medication use, which as a result, has potential for medicines related problems (MRPs) and harm.^7^ Undoubtedly, unchecked MRPs can lead to deterioration in health and death.^8^ Medicines, medication, or drug related problem (DRP) are terms that have been used in different studies to refer to the same concept.

An MRP or a DRP as referred to by Hepler & Strand (1990)^8^ is defined as “an event or a circumstance involving drug treatment that actually or potentially interferes with the patient experiencing optimum outcome of medical care.”^8^ On the other hand, deterioration in health has been defined as clinical decline or onset of sudden acute episodes of ill health.^9^ Hence falls, delirium, increased use of health care services, and hospitalization which can culminate in death can be considered as markers of deterioration in health. Studies indicate that certain MRPs are associated with increased use of health care services and hospitalization in older people.^10,11^ Similarly, clinical decline in older people, has been shown to result in hospitalization more so when the events are of sudden or acute onset.^12^ Indeed, results from one published systematic review suggested that the prevalence of hospitalization from MRPs can be up to 12.1%.^13^ The implication of these findings is that MRPs and clinical decline can be associated with hospitalization of affected individuals. Sadly, hospitalization often translates to some form of cost to the patient as well as translating to an economic cost that can be worrying for any health care system.^14^ Thus, the annual cost of hospital admissions associated with one type of MRPs, notably adverse drug reaction (ADR), was estimated to be £466 m (US $847 m) by an English study undertaken over a decade ago.^11^ Similarly, a rapid evidence synthesis and economic analysis of studies published about a decade ago, estimated the cost of avoidable Primary care ADR to be £83.7 m (US$150.66 m) per annum.^14^ In terms of global estimate, worldwide, the annual cost associated with medication errors (one other type of MRPs) was £23 billion (US$42 billion).^15^ This constituted almost 1% of the total global health expenditure.^15^ It would be appropriate to suggest that the cost associated with MRPs makes the study of MRPs and of the preventative interventions to mitigate it imperative. This is a global necessity, given both an increasing age demographic and scarce resources, more so since events due to MRPs are potentially preventable.^14-16^ Importantly, most medicines related problems that can lead to hospitalization resulting in a cascade of problems within hospitals have their origin within primary care setting.^17^ Hence it is fitting to have this study on MRPs originating in Primary care. A number of systematic reviews have explored aspects of medicines related problems occurring outside secondary care settings and leading to hospitalization and /or death. Others, however reported prevalence of MRPs without health outcome associated with it. Thus, a systematic review by Morin, Laroche, Taxier and Johnell (2016)^18^ reported a 43.2% weighted point prevalence of potentially inappropriate medicine (PIM) in nursing home. However, it did not explore its impact on health of older adults, an assessment important for health-related economy. Another systematic review reported a higher rate of hospitalization in nursing home residents exposed to PIM when compared to those in the community.^19^ Similarly, there are published systematic reviews on interventions to improve polypharmacy in older people.^20,21^ Hence it can be right to say that several works including systematic reviews had been undertaken on medicines related problems in older people. However, none to the knowledge of the reviewers explored within one systematic review several MRPs occurring in older people arising in primary care and indicators of deterioration that can potentially be associated with them as explored in this review. This is important given that a cohort of patients can often present with different types of MRPs leading to hospitalization, use of other health services or death. Furthermore, systematic review is considered a gold standard for evidence-based practice.^22^

**Aim:** To explore published primary studies on MRPs occurring in older people from Primary health care settings, including where this has led to hospitalization; to explore the types of tools/interventions employed to identify and prevent MRPs in this patient group.

**Review Objectives:** To ascertain from published studies, medicines related problems associated with priorly determined indicators of deterioration, most frequently affected patient group reported, the residential settings of these patients, risk factors, tools employed to identify MRPs, and interventions applied to prevent MRPs

**Methods:** The scope of the review was defined by applying the acronym PICOS (Population, Intervention/Exposure, comparison, Outcome, Setting).^23^ Subsequent to this, though a protocol was not registered, a systematic review plan guided by PRISMA-P (Preferred Reporting Items for systematic review for protocols) was drawn up and approved by the research team prior to the commencement of the systematic review.^24^ The plan was employed as a guidance document to systematically review relevant Primary studies published between 2001 and 2018. It described the scope, rational, intended purpose, as well as the methodological and analytical approach to the review. Ethical approval was not required prior to commencement of review as the use of patients’ identifiable data was not intended.

## Inclusion Criteria

The agreed indicators for deterioration were fall, delirium, pressure ulcers, hospitalization, use of health services and death, chosen because of the association of these indicators with older people.^4,5^ Selected studies were assessed against the following inclusion criteria: (i) Studies written in English language; (ii) Population of people aged 65 years and over; (iii) with an exposure to or intervention for medicines related problems originating in Primary care;(iv) with outcomes- hospitalization, use of health services, falls, delirium, pressure ulcers or death; (v) Primary studies (vi) Published between 2001 and 2018. Includable study designs were observational (retrospective or prospective case control, case series non- interventional, cross sectional, cohort) and interventional (quasi-experimental, randomized controlled trials, case series interventional) studies. Studies from year 2001 were included in this review to make the included studies contemporary with the UK’s initiatives on medicines management for older people.^25^ This was an initiative, included in the first official document on health and social care for older people in the UK (National service framework for older people). Put succinctly, it was about using right medicines in the right context (people and time). Although this document was initiated in the UK, it has global relevance as medication use and old age are common issues globally.

## Exclusion Criteria

Any study that did not fulfill criteria for inclusion, studies unrelated to review objectives, abstract -only papers, none-human subject studies, systematic review studies.

### Data Sources and Search Methods

Electronic search of International Pharmaceutical Abstracts, MEDLINE (via PubMed), CINAHL, Embase, Psych INFO, PASCAL, SCOPUS, Cochrane Library, Science Direct and Zetoc as well as references within included articles were undertaken for relevant studies (Table 1). Choices of data bases to be searched were based on insights gained from the method’s section of related reviews. All data bases were searched from 2001 to March 2018.

**Table 1.** Data Base and Terms Employed to Obtain Relevant Studies.

Data bases searched (From 2001 - March 2018)	Terms used for searching in medline
MEDLINE via PubMed MEDLINE via PubMed Cochrane Scopus CINAHL Zetoc Embase Psych INFO PASCAL International Pharmaceutical Science Direct	Population: Older OR elderly OR (older people) OR (older person) OR (older population) OR (elderly people) OR (elderly person) OR (elderly population) AND Intervention/Exposure: Medicines Related Problems (MRPs) OR (Adverse drug event) OR (adverse drug reaction) OR (inappropriate medi$) OR (pharmaceutical care issues) OR (pharmaceutical services) OR (medication error) AND Outcomes events: (Pressure ulcer) OR (geriatric syndrome) OR delirium OR fall OR fracture OR hospitali? ation OR death AND Setting: (Primary care) OR (Primary health care) OR (general practice) OR (family practice) OR (patient admission) OR (patient discharge) OR (continuity of patient care) OR (doctor’s office) OR (ambulatory care) OR (accident and emergency) OR surgery

MRPs, medicines related problems, medication error, adverse drug event, adverse drug reaction, non-adherence, potentially inappropriate medicines, and pharmaceutical care issues, identified within the MEDLINE database through the MeSH term “pharmaceutical services,” were employed as search terms for MRPs. Falls, delirium, pressure ulcers, hospitalization, use of health services, and death were employed as search terms for indicators of deterioration. Settings were specified as primary care, general practice, family practice, care homes, community, patient admission, and patient discharge.

Boolean operators, AND/OR were used to combine search terms. The “snowballing” strategy, going through the reference list of all included studies to obtain further relevant studies was also employed^26^

### Study Selection and Validation Process

Following a literature search of the databases by one reviewer (RO), studies were exported to Endnote X7. Titles and abstracts were screened for relevance, duplicates were removed followed by screening the complete articles for possible inclusion by one reviewer (RO). Another reviewer, (NU) independently reviewed the titles, abstracts, and full studies, confirmed relevance of studies in meeting the inclusion criteria and excluded studies deemed to be irrelevant. There was complete agreement on relevance of selected studies by RO and NU.

## Methodological Quality (Risk of Bias Assessment)

A quality assessment checklist proposed by Downs and Black for the methodological quality of randomized and non-randomized studies of health care interventions was adapted for this review.^27^ It is a commonly used and well validated rating scale.^28,29^ The original scale assigns a total score out of 32 points. In line with previous studies, a modified version of the scale was employed by simplifying the power question and awarding a single point to studies with sufficient power to detect a clinically important effect, where the probability value for a difference being due to chance is <5%.^30^ The review team acknowledged that statistical significance does not always equate to clinical significance.^31^

Though ROBINS-1 is usually, the preferred tool for assessing risk of bias in non-randomized trials, the decision on choice of tool for this review was based on the included studies comprising of both randomized and non-randomized studies.

### Data Extraction and Synthesis Process

Data extraction forms were created consisting of study design, setting, mean age, sample size, MRPs, outcome deterioration, implicated medicines, risk factors and reported interventions presented in Tables 2 and 3, respectively. Three studies were initially piloted to test the forms. Data was extracted into these forms. Data obtained from similar research settings were grouped together and summarized using narrative synthesis. Meta-analysis could not be performed because of the heterogeneity of the included studies.

**Table 2.** Characteristics of Included Studies on MRPs.

Study design Country	Setting age (in years)	MRPs type methods of identification prevalence	Indicator of deterioration/rate of medicines related hospitalization	Number of indicators associated with MRPs	Medicines implicated in MRPs	Risk factors recorded Most affected patient group
Barber et al (2009)^47^ Mixed method prospective study UK	Care home Mean age 85	Medication error identified via interviews and observations prevalence:69.5%	No specific indicator but potential for and cause of harm from MRPs was identified	0	ACEI (angiotensin- converting enzyme inhibitors)	High workload inadequate knowledge Lack of teamwork Inefficient ordering system Inaccurate medicines records Prevalence of verbal communication
Barnett et al (2011)^41^ Retrospective cohort study UK	Care home and Own home Mean age 75.2	Potentially Inappropriate medicines (PIM) Identified by the Beers Criteria Prevalence of PIM: 20-46%	Death	0	Long acting benzodiazepines Fluoxetine Muscle relaxants Nitrofurantoin Amitriptyline NSAIDs	Living in care, Older age, and additional polypharmacy
Lau et al (2005)^50^ Analysis of retrospective longitudinal data USA	Care home (Nursing home) Mean age 85	PIM Identified by Beers Criteria	Hospitalization Death PIM related = 33%	2	Antihistamine, oxybutynin chloride, amitriptyline hydrochloride, iron supplement, ranitidine	Intermittent PIM
Gurwitz et al (2005)^16^ Prospective case control study Canada	Care home Mean age 86 ± 8	Medication error, ADR. Prevalence of ADR 9.8 per 100 resident months	Delirium death fall	3	warfarin Atypical antipsychotic loop diuretic opioids antiplatelet ACEI	Polypharmacy
Budnitz et al (2011)^49^ Secondary data analysis. USA	Hospital Mean age ≥ 65	ADR Unintentional overdose PIM Identified by Beers criteria	Hospitalization ADR related = 37.5%	1	warfarin Insulin Oral antiplatelet Oral hypoglycaemic	Polypharmacy
Endres et al (2016)^36^ Prospective cohort study Germany	Hospital Mean age ≥ 65	PIM Identified by PRISCUS list Prevalence: 23.5%	Hospitalization PIM related = 6%	1	Benzodiazepines Analgesics Antidepressants Muscle relaxants Antihypertensives	Polypharmacy
Hofer-Dueckelmann et al (2011)^35^ Prospective observational study Austria	Hospital Mean age, 66.5 ± 15.8	ADR Prevalence: 7.6%	Hospitalization ADR related = 7.6%	1	Diuretic Vitamin K antagonist ACEI NSAID Beta blockers	Impaired renal function, Polypharmacy cognitive impairment, need for care, female gender
Howard et al (2008)^48^ Qualitative case studies UK	Hospital	Medication error Identified using a framework of Reason’s model	Hospitalization	1	Not reported	communication problems, Knowledge gap
Laatikainen et al (2016)^38^ Retrospective study Finland	Hospital Mean age ≥ 65	ADR Prevalence MR- hospitalization 23.1%	Hospitalization falls delirium ADR related = 23.1%	3	Opioids, benzodiazepines levodopa, memantine isosorbide mononitrate, carbamazepine,	Poly pharmacy
Leendertse et al (2008)^37^ Prospective multicentre study Netherlands	Hospital	ADR medication error prevalence MR-hospitalization 5.6%	Hospitalization ADR related = 5.6%	1	Antiplatelets diuretics insulin oral antidiabetic β-Blockers	Impaired cognition ≥4 comorbidities dependent living renal impairment nonadherence to medication regimen polypharmacy
Pirmohamed et al (2004)^11^ Prospective analysis UK	Hospital Age range was 65-83 years	ADR Prevalence of ADR = 6.5%	Hospitalization Death ADR related = 6.5%	2	NSAID Diuretics Warfarin ACEI Antidepressants	Medication interaction
Van der Stelt et al (2015)^45^ A nested case control study Netherlands	Hospital Age range was ≥ 65	PIMs and PPO (potential prescribing omission) Identified by Beers criteria and by STOPP/START criteria Prevalence PIM = 34.1% to 44.4% PPO = 57.7%	Hospitalization PIM related = 5.6%	1	NSAIDs Benzodiazepine	Impaired cognition, polypharmacy ≥3 comorbidities, renal impairment
Wierenga et al (2012)^5^ Prospective cohort study Netherlands	Hospital Mean age: 77.8	ADR	Hospitalization Delirium (25.9%) Falls (12%) ADR related = 12%-25.9%	3	Diuretics Prednisolone NSAID Antidepressant Antipsychotic	Co-morbidity Functional impairment Cognitive impairment
Beer et al (2010)^34^ Prospective observational cohort study Australia	Community Mean age: 77 ± 3.6	PIM According to Australian criteria for assessment Prevalence: PIM = 48.7%	Fall Hospitalization Death	3	NSAIDs, Allopurinol Antihypertensives Benzodiazepine Digoxin Tricyclic antidepressants, Antihistamine	Polypharmacy Underutilization of medicines
Cahir et al (2014)^40^ Retrospective study Ireland	General practices Mean age:78	PIM Identified by STOPP criteria ADR Prevalence: PIM = 42%	Use of health services	1	anti-thrombotic anti-inflammatory psycholeptics psychoanaleptics	Number of different repeat drug classes, medication possession ratio (a measure of medication adherence)
Henschel et al (2015)^39^ Retrospective analysis Germany	Age range ≥ 65	PIM Identified with the PRISCUS list	Hospitalization PIM related = 10%	1	Analgesics Cardiovascular drugs Antibiotic Antidepressants Antiplatelet Sedatives	Greater age Higher co-morbidity Preceding events of hospitalization

**Table 3.** Characteristics of Included Studies on Interventions for MRPs.

Study design Country	Setting age (in years of participants)	Intervention	Follow up	Outcomes	Relevant findings
Chan et al (2014)^46^ prospective case- series intervention study Taiwan	Outpatient department Mean age 75.6 ± 6.1	Medication safety review clinic (MSRC) for solving drug related problems (DRPs) among older adults prescribed multiple medications	24 weeks	Patients who had at least one unsolved DRP Changes in the number of total medications Changes in physical functioning Patients satisfaction between weeks 1 and 24	40 unsolved DRPs at week 24. The mean number of chronic medications decreased by 0.4 Unexpected functional decline of 0.5 points Percentage of participants rating their general health as good or better, increased from 22% to 38% in 24 weeks
Gallagher et al (2011)^10^ A randomized controlled trial UK	Hospital Cork University Hospital Age range ≥65years	Screening of hospitalized older patients’ medication against STOPP/START criteria for PIM/PPO and providing recommendations to the attending medical team	6 months	Medication appropriateness index (MAI) Frequency of unnecessary polypharmacy Prevalence of fall Prevalence of all-cause mortality Length of hospital stay Rate of PIM assessed using STOPP/START criteria	Lower MAI scores at discharge than at admission (absolute risk reduction 35.7%) Reduction in unnecessary polypharmacy (from 20.0% at admission to 5.4% at discharge Non-significant reduction in falls and in all-cause mortality during 6-month follow up STOPP/START PIM increased gradually during the follow up period in both groups
Gillespie et al (2009)^43^ A randomized controlled trial Sweden	Hospital University Hospital with follow up in community Age range ≥ 80 years Follow up: 12 months	Interventions provided by ward-based pharmacist: Medication reconciliation Performance of drug review Provision of advice to patient’s physician Patients education and monitoring, Patient counseling at discharge Follow up telephone call two months after discharge	12 months	Frequency of hospital visits during a 12-month follow up period	Intervention group vs control group: 16% reduction in all visits to the hospital and a 47% reduction in visits to the emergency department Drug related readmissions were reduced by 80%
Sellors et al (2003)^42^ A randomized controlled trial Canada	Primary care 24 sites of family practices Age range ≥ 65 years	Pharmacists conducted face-to-face medication reviews with patients in the intervention group. Then gave written recommendations to the physicians Hepler and Strand definitions of DRPs was used	5 months	Number of DRP identified in the intervention arm Proportion of the recommendations implemented by the physicians	Mean of 2.5 DRPs identified in the intervention group No statistically significant difference in number of medicines, or health care use between groups Physicians implemented/attempted to implement 72.3% of recommendations.
Wang et al (2013)^44^ Randomized control study Taiwan	Community Age range ≥ 65 years	Medication safety program including a coach, and reminders by well-trained volunteers, two home visits and five telephone calls over a two-month period Both groups received routine medication safety instructions for their chronic illness	2 months	Medication safety knowledge Medication safety attitude	After two months of coaching the intervention group demonstrated higher scores than the control group with regard to medication safety knowledge but no significant difference in attitudes scores between two groups

It is necessary to note here that since adverse drug event (ADE) was not categorized in the Hepler and Strand 1990 classification of MRPs unlike ADR, for this review an ADR will refer to either ADE or ADR.^8^ An adverse drug event is defined as an injury resulting from medical intervention related to drug(s).^32^ On the other hand, ADR is “an appreciably harmful or unpleasant reaction, resulting from interventions relating to the use of a medicinal product which predicts hazard from future administration and warrants prevention or specific treatment or alteration of the dosage regimen, or withdrawal of the product.”^33^ This is a comprehensive definition that can apply both to adverse drug events and reactions.

## Results

**Figure 1.**
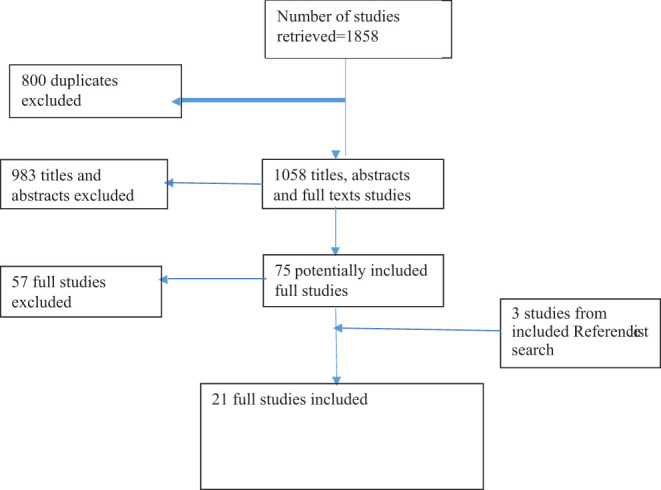
The PRISMA flow diagram for study selection.

Figure 1 provides an overview of the search and selection process. A total of 1858 studies were identified, of which 21 studies satisfied the inclusion criteria. The included studies consist of 16 studies on deterioration due to MRPs and 5 studies on interventions to prevent deterioration due to MRPs. The included studies consisted of six prospective observational studies Australia,^34^ Austria^35^ Germany,^36^ Netherlands,^5,37^ UK.^11^ Four retrospective cohort studies, Finland,^38^ Germany,^39^ Ireland,^40^ UK.^41^ Four randomized controlled trials, Canada,^42^ Sweden,^43^ Taiwan^44^ UK.^8^ Two case control studies, Canada,^14^ Netherlands.^45^ One prospective case series intervention, Taiwan.^46^ One mixed methods study UK.^47^ One qualitative case study, UK.^48^ Two secondary data analysis, USA.^49,50^

The studies were conducted in three types of settings: Care home^16,47,50^ Hospital (where MRPs originated from primary care)^11,35,37,38,45,48^ and Community (with type of community setting unspecified).^34,40^ A UK retrospective cohort study^41^ included participants from care homes and their own homes but for this review is reported under care home. On the average, patients were aged 75 years based on 15 studies.

Methodological Quality of studies: Table 4: Assessment of methodological quality for 20% of included studies was achieved using adapted tool.^27^ All studies were rated and assigned a grade of “good” (19-23 points).

**Table 4.** Assessment of Methodological Quality of Studies.

Serial number	Item/study	Gurwitz et al, 2005^16^	Barber et al, 2009^47^	Barnett et al, 2011^41^	Pirmohamed et al, 2004^11^	Chan et al, 2014^46^	Gillespie et al, 2009^43^
1	Hypothesis/aim/objective	Yes = 1	Yes = 1	Yes = 1	Yes = 1	Yes = 1	Yes = 1
2	Outcomes	Yes = 1	Yes = 1	Yes = 1	Yes = 1	Yes = 1	Yes = 1
3	Patients characteristics described	Yes = 1	Yes = 1	Yes = 1	Yes = 1	Yes = 1	Yes = 1
4	Interventions described	Yes = 1	Yes = 1	Yes = 1	Yes = 1	Yes = 1	Yes = 1
5	Confounders described	No = 0	No = 0	No = 0	No = 0	N0 = 0	No = 0
6	Study findings described	Yes = 1	Yes = 1	Yes = 1	Yes = 1	Yes = 1	Yes = 1
7	Estimate of random variability described	Yes = 1	Yes = 1	Yes = 1	Yes = 1	Yes = 1	Yes = 1
8	Important adverse events reported	Yes = 1	No = 1	Yes = 1	Yes = 1	Yes = 1	Yes = 1
9	characteristics of patients lost to follow up reported	No = 0	No = 0	Yes = 1	No = 0	Yes = 1	Yes = 1
10	Actual probability reported	Yes = 1	No = 0	No = 0	Yes = 1	Yes = 1	No = 0
11	Representative sample obtained	Yes = 1	Yes = 1	Yes = 1	Yes = 1	Yes = 1	Yeas = 1
12	Proportion of those asked who agreed to participate stated	Yes = 1	Yes = 1	Yes = 1	Yes = 1	Yes = 1	Yes = 1
13	Sample drawn from a setting representative of majority population	Yes = 1	Yes = 1	Yes = 1	Yes = 1	Yes = 1	Yes = 1
14	Attempts made to blind study subject	Yes = 1	Yes = 1	Yes = 1	Yes = 1	No = 0	Yes = 1
15	Attempts made to blind those measuring outcomes	No = 0	No = 1	No = 0	No = 0	No = 0	No = 0
16	Was any data dredging reported	Yes = 1	Yes = 1	Yes = 1	Yes = 1	Yes = 1	Yes = 1
17	In cohort studies do analysis adjust for length of follow up	Yes = 1	Yes = 1	Yes = 1	Yes = 1	Yes = 1	Yes = 1
18	Appropriate statistical test used to measure main outcome	Yes = 1	Yes = 1	Yes = 1	Yes = 1	Yes = 1	Yes = 1
19	Compliance with intervention reliable	Yes = 1	Yes = 1	Yes = 1	Yes = 1	Yes = 1	Yes = 1
20	Main outcome measures used accurate	Yes = 1	Yes = 1	Yes = 1	Yes = 1	Yes = 1	Yes = 1
21	Patients in different intervention group similar	Yes = 1	No = 0	Yes = 1	Yes = 1	Yes = 1	Yes = 1
22	Recruitment to different intervention group over the same period	Yes = 1	Unable to determine = 0	Yes = 1	Yes = 1	Yes = 1	Yes = 1
23	Randomization of study subject done	No = 0	No = 0	No = 0	No = 0	No = 0	Yes = 1
24	Concealment of randomized interventions	No = 0	No = 0	No = 0	No = 0	No = 0	Yes = 1
25	Adequate adjustment for confounding	No = 0	No = 0	No = 0	No = 0	No = 0	No = 0
26	Loses of patient to follow up taken into account	Unable to determine = 0	Unable to determine = 0	Unable to determine = 0	Unable to determine = 0	Yes = 1	Unable to determine = 0
27	Sufficient power	Yes = 1	Yes = 1	Yes = 1	Yes = 1	Yes = 1	Yes = 1
	Total	20	18	20	20	21	22

### MRPs in Care Home Setting (Including Nursing and Residential Homes)

The reported prevalence of MRPs was 9.8% to 69.5%. The MRPs reported were Potentially Inappropriate Medicines (PIM),^41,50^ Medication error (ME)^47^ and adverse drug reaction (ADR).^16^

Two studies examined the association between PIM and risk of hospitalization and death. The Beer criteria which is a tool by American Geriatric Society for identifying potentially inappropriate medication use in older adult was used. Descriptive statistics was used to establish associations between PIM and risk of hospitalization and death^50^; lack of association between PIM and death but an association between number of medicines and death.^41^ According to findings from one study, the risk of hospitalization was 33% and risk of death was 28% greater for residents with PIM exposure when compared to those without.^50^

One study examined ME and reported the potential for harm through prescribing, monitoring, administration and dispensing errors.^47^ One study reported ME as a cause of ADR resulting in delirium, fall, fall with fracture and death.^16^ Types of drugs implicated in MRPs were cardiovascular drugs^16,47^ Anticoagulants, antipsychotics, and long-acting benzodiazepines.^16,50^ Other implicated medicines identified by single studies were anti-infectives and antiepileptics,^16^ antihistamine with strong anticholinergic effect, narcotics, antispasmodic agents, and iron supplements^50^

The most at-risk groups were those taking drugs from several drug classes^16^; patients experiencing intermittent use of PIM^50^; patients on polypharmacy.^16,41^

### MRPs in Hospital Setting

The reported prevalence of MRPs presented at hospitals from primary care was 6.5% to 48.7%. The MRPs reported were PIMs,^36,39,45^ Potential prescription omission (PPO),^45^ ADR,^5,11,35,37,39,49^ unintentional overdose^49^ and ME.^5,37^

Two studies examined association between PIM and risk of hospitalization. These two studies used PRISCUSS List which was developed specifically for use in Germany to identify PIM in the elderly.

Multivariate regression was used to establish association.^36,39^ One study examined association between PIM/PPO and risk of hospitalization, it used STOPP (Screening Tool of Older persons Prescription for identifying potentially inappropriate medicines)/START (Screening Tool to Alert doctors to Right Treatment for identifying potentially prescription omission) criteria 2008 to identify PIMs and PPOs, similarly, multivariate regression was used to establish association.^45^ However, when the same study employed Beers criteria 2012 to identify PIM, it found no association between PIM and risk of hospitalization. Though, the presence of two or more PIMs identified with Beers 2012 and STOPP 2008 was associated with a higher risk of hospitalization than the detection of no PIMs.

Two studies examined association between ADR and medication related hospital admission. These studies employed Naranjo algorithm (an algorithm for determining that an adverse drug event is due to drug rather than to other factors) to establish association.^11,35^

Three studies reported associations between ADR and hospitalization. One employed the Algorithm of Krammer (an algorithm for ranking probability of causation between a drug and a clinical manifestation in an adverse drug reactions).^37^ A second study employed clinical pharmacist and geriatrician assessment.^5^ The third study was by physicians’ diagnosis.^49^ Two studies identified ME as the cause of ADR.^5,37^ Two studies identified death as a consequence of medicines related hospitalization^11,37^

The classes of medicines most implicated in the reasons for hospitalization were non-steroidal anti-inflammatory drugs (NSAIDs),^5,11,35,37,45^ antiplatelet drugs,^11,37,49^ diuretics,^5,11,35^ oral anticoagulants,^11,37,49^ antidiabetic drugs^37,49^ and angiotensin converting enzyme inhibitors.^11,35^

Groups most at risk of hospitalization from MRPs were older people with polypharmacy, impaired renal function, care needs, mental impairments as well as women.^35,37,39^ Similarly, those with comorbidities were reported to be at risk.^5,37,45^

### MRPs in Community Setting

The MRPs reported were ADR,^40^ PIM^34^ and ME^48^

Three single studies examined the association between PIM and ME respectively with hospital admissions^34^ use of health services^40^ and Medicines Related Hospital Admissions.^48^These studies gave conflicting results which were that admission for fall and for geriatric syndrome were not independently associated with any markers of suboptimal prescribing.^34^ However, number of medicines and use of one or more PIM were independently associated with all cause admission to hospital and greater hazard of admission to hospital.^34^ Similarly, active failure in medication use process including prescribing, dispensing, administering, and monitoring resulted in drug related hospital admission.^48^ Patients with two or more PIP were twice as likely to have an ADR and have nearly a twofold increased risk in the expected rate of A&E visit^40^

### Studies on Interventions to Stop MRPs

Studies were undertaken in Canada, Sweden, Taiwan, and UK. Reported interventions comprised education of service users and medication review with therapeutic recommendation (Table 3).

Interventions employed in two studies were education of service users (Taiwan)^44,46^ and service providers.^46^ Coaching rural Taiwanese elders on medication safety^44^ resulted in an improvement in medication safety behavior but no apparent attitude change. However, education of service providers (prescribers) and service users (patients)^46^ indicated a reduction in the total number of chronic medication prescribed as well as participants’ better report on self-health.

Three studies were on medication review with therapeutic recommendation, Canada,^42^ Sweden^43^ and UK.^10^ Medication review and reconciliation in addition to patient education, and therapeutic recommendation to the prescriber resulted in a 16% reduction in all visit to the hospital, 34% reduction in visit to the accident and emergency, and an incremental cost saving.^43^ Applying STOPP/START screening tool followed by recommendations to patient’s doctor demonstrated a reductions in unnecessary polypharmacy, inappropriate medicines, prevalence of falls, all-cause mortality and MRPs with an absolute risk reduction of 35.7%.^10^ On the other hand a study with a relatively small sample size of pharmacist intervention targeting polypharmacy in patients with a broad variable health status yielded neither a reduction in polypharmacy nor an improved health outcome.^42^

## Discussion

There is a variation in the prevalence of MRPs reported by different studies which can be attributed to a number of factors: Notably, there were variations in types of MRPs, denominator or population used for calculation, types of tools used for identification, as well as what data was available to the researchers.

For instance, Endres et al (2016),^36^ to calculate prevalence of PIM used as a denominator 392,337 ambulatory patients aged 65 years and over, who had routine claims data between January 2009 and December 2010. There was a variation in prevalence of PIM within this study that ranged from 19.3% to 58.4% depending on the part of year for which PIM was calculated. Their identification tool was the PRISCUS List. On the other hand, Barnet et al (2011),^41^ used as a denominator 70299 cohort of older people who were 66 years and over, lived in either care home or in own home. They reported a PIM prevalence of 37.1% for care homes and 30.9% for own homes, not for a particular period in the year. The findings from these two studies indicates that those in care (Care home or nursing homes) can be more at risk of receiving PIM than those that are not in care (ambulatory patients in their own home). Secondly, the time of year may have a role in the risk of exposure to PIM as identified by Endres et al.^36^ Thus, time of year can potentially be a confounding factor in exposure to MRPs^51^ (Pazzagli et al, 2018) Finally, Barnet et al (2011)^41^ acknowledged that assumptions were made when they encountered missing data. These assumptions could have impacted on the calculated prevalence. They identified PIM using Beer’s Criteria.

The prevalence of ADR related admission was different for the different studies. Thus, Pirmohamed et al (2004)^11^ reported a prevalence of 6.7% while Laatikainen (2016),^38^ reported a prevalence of 23.1%. It is worth noting that while these two studies reported prevalence of events (hospitalization) from MRPs the other two studies reported prevalence of MRPs (PIM). Hence prevalence rates would be different.

However, despite significant variations in study designs and results a clearer picture emerges: Medicines Related Problems (MRPs) occur across the Primary health care. Across studies, number of indicators of deterioration associated with MRPs were between 1 and 3. These include, falls, delirium, use of health services hospitalization and death. In care homes, common MRPs reported were Medication error at a prevalence of up to 69.5%, potentially inappropriate medicines (PIM) at a prevalence of 20-46%, and adverse drug reaction at a prevalence of up to 9.8% per 100 resident months. Residents with PIM had 33% greater risk of hospitalization and 28% greater risk of death than those without. The prevalence of PIM among patients presenting from primary care to hospital was between 23.5% to 48.7%, while that of ADR was between 6.5% to 7.6%. Findings from other empirical studies and from systematic reviews suggest that PIM in care homes is a longstanding and global problem that deserves attention.^18,52,53,54^ The reported prevalence of hospitalization associated with MRPs was 5.6% to 33% for PIM and 5.6% to 37.5% for ADR. This suggests that the prevalence of hospitalization from MRPs can be similar across types of MRPs. Errors in prescribing resulted in potentially inappropriate medicines.^55^ Similarly, for an older person PIM can result in adverse drug reaction.^55,56^ This can thus present a cycle of inappropriateness in medication use for older people.

Interestingly, findings from two of the included studies presented conflicting results in terms of the relationship of PIM in care homes with death and hospitalization. Hence one study suggested that both hospitalization and death were associated with PIM.^50^ On the other hand, another study suggested that no relationship existed between all cause death and PIM.^41^ There are two possible explanations for this, while the participants in the study that recorded an association were residents in nursing homes,^50^ those in the study that recorded no association were from care homes, nursing homes or from their own homes.^41^ The participants from nursing homes, can be said to be sicker and hence more vulnerable to the effect of PIM than participants who were from care homes and their own homes^57^ Secondly, there were differences in the types of medicines implicated in PIM as studies were conducted in two different countries, UK, and US, respectively. Studies have indicated that not all medicines contained in the Beers criteria have relevance in the UK^58^

Undoubtedly, medication error including PIM is a source of problem for older people in care homes^59,60^ as well as in other settings in the primary care. Inappropriate prescribing is a risk for medication error and for adverse drug reaction which can potentially result in deterioration of health indicated by hospitalization.^61^ However, the fact that certain tools like STOPP/START, the Beers criteria and the PRISCUS List have been used by researchers and Clinicians in certain settings to identify MRPs suggests that applying these tools in routine practice can be achieved.^39,62^ The relevance of such practice would be to support appropriate prescribing hence prevent and reduce potential deterioration from medicines related problems. In the UK for instance, the national institute of care and health excellence (NICE) recommends the use of STOPP/START to support prescribing in order to avoid inappropriate polypharmacy.^63^

Similar to the findings from this study, that from other single studies (Bohlken and Kostev, 2018; Onder et al, 2018)^64,66^ not includable in this review, also suggest that delirium is associated with medication use^64,65^ and that delirium is a risk factor in falls.^66^ Therefore, the implication for a patient that has MRPs associated with delirium is an increased risk of falls. It is therefore correct to suggest that indicators of deterioration which are, death, delirium, fall, hospitalization and use of health services explored in this systematic review can be associated with MRPs.

Reported risk factors for MRPs were drug use from several drug classes, intermittent use of PIM and polypharmacy. Reports from other single studies suggest that polypharmacy is a common problem in care homes globally.^65,67,68^ Though, the terminology employed in defining polypharmacy can vary, what is important is not the number of medicines but the appropriateness of the medicines.^69^ However, with an increase in number of medicines there is a greater opportunity for an inappropriate medicine.^41^ In addition to polypharmacy, the other risk factors for hospitalization from MRPs were age, number of comorbidities, being a woman and dependent living. Hence a comorbid older patient, with polypharmacy, living dependently, if exposed to MRP, will be at a greater risk of hospitalization.^70^

The classes of medicines implicated in MRPs were antiplatelets, diuretics, NSAIDs, ACEIs, anticoagulants, and hypoglycemics. These are similar to those reported in a review carried out over a decade ago.^71^ The fact that following on from a decade ago, the same classes of medicines are implicated in MRPs can suggest that monitoring of medication use in older people needs to be intensified. Finally, narcotics, antihistamines with strong anticholinergic effects, antispasmodic agents, iron supplements, anti-infectives and anti-epileptics though not widely reported in other reviews were identified as implicated in MRPs by this review. This finding is important because attention is required in planning interventions for older people.

Tools to identify PIM were Beer’s criteria, STOPP/START criteria, and the PRISCUSS List. These are clinical tools developed to assist health care providers in providing safe medicines to older people. However, the outcome from applying these tools proved to be context and country related. In addition to this, the extent to which these tools are applied to support medication use in primary care is questionable.

Furthermore, preventative interventions to mitigate MRPs, were mostly successful variations of medication review. Hence a medication review clinic resulted in a 0.4% reduction in mean number of medicines and an improvement in general health ratings of participants from 22% to 38% over a six-month period.^46^ Similarly, a study that applied STOPP/START to identify PIM/PPO coupled with recommendation to a medical team, reported a reduction in unnecessary polypharmacy from 20.0% at admission to 5.4% at discharge. However, there was a non-significant reduction in falls and in all-cause mortality during the 6 months follow up. STOPP/START PIM increased gradually during the follow up period in both groups.^10^On the other hand, a comprehensive medication reconciliation intervention with education and monitoring of the intervention group and a two month follow up, resulted in an 80% reduction in drug related admissions.^43^ The implication is that the intervention that worked well were those that were comprehensive with an element of patients’ education in addition to an ongoing conversation with patients.

The systematic review highlights the need for prescribers, usually medical doctors to reassess prescribing habits and use appropriate decision tools to optimize prescribing for older people. Similarly, Pharmacists can apply this tool in optimizing the medication review process. In addition, pharmacists require collaborations with Doctors and patients to achieve success in medication review interventions.

Finally, increase in risks of MRPs with increase in the number of medicines is of particular relevance to older people who usually take multiple therapies due to multiple co-morbidities. Hence the WHO (World Health Organization)^15^ recognized Polypharmacy as a priority area in medication safety and in its mandate “medication without harm” advocates appropriate interventions to address this globally.

Pharmacists working in collaboration with other health care professionals have an important role to play in this global mandate.^8^

The importance of study finding:

- Reliability of identification tools for PIM can be country specific according to the included drugs list of the country. It is important that appropriate tools are used in each country to achieve reliable outcomes
- Presently, there is considerable variations in reported outcomes when different tools were employed to investigate PIM. There is thus a need to standardize systems as this will allow better comparison of outcomes and planning of effective interventions
- Reduction in the occurrence of medication errors in primary care can potentially result in the reduction MRP outcomes.
- Collaboration among health care professionals and Patients in planning and executing MRP interventions is required for optimal results.
- Comorbidity, cognitive impairment, and polypharmacy appear to be common in people hospitalized due to MRPs.
- Patients that present with indicators of deterioration such as falls, delirium, as well as those who without these, require health care services or hospitalization should be assessed for medicines related problems. This can prevent further deterioration if adequate intervention is offered such patients.

### Limitations of the Systematic Review

Searching only published data bases could have resulted in missing out some potentially relevant but unpublished studies from the review. Secondly limiting to studies published in English language could have resulted in missing important studies published in other languages.

### Limitations of the Evidence

To the knowledge of the authors, this is the first systematic review to assess medicines related problems associated to potential indicators of deterioration. However, the quality of available evidence to link these indicators to deterioration in health are relatively weak. Variation in tools used for identification of MRPs limited comparability of findings across studies.

### Comparison with Existing Literature

A few reviews have assessed MRPs in hospitalized patients. However, none to the knowledge of the reviewers have focused on MRPs originating in Primary care and associated with potential indicators of deterioration of health as explored in this present systematic review.

### Implication for Research and Practice

A few studies identified as outcomes, the indicators of deterioration which are relevant to this systematic review. Therefore, validating the concepts of deterioration in subsequent studies is required as this will provide measurable indicators for assessing its prevalence. It will also enable the planning and evaluation of interventions. Secondly, further research is required to develop methods for standardized measurements for medicines related problems that will allow greater comparability of outcomes across studies. Lastly, there was evidence that medication safety interventions are best achieved with inputs from all relevant stake holders and that pharmacists have a role to play in this.
